# Heuristic machinery for thermodynamic studies of SU(*N*) fermions with neural networks

**DOI:** 10.1038/s41467-021-22270-5

**Published:** 2021-03-31

**Authors:** Entong Zhao, Jeongwon Lee, Chengdong He, Zejian Ren, Elnur Hajiyev, Junwei Liu, Gyu-Boong Jo

**Affiliations:** 1 Department of Physics, The Hong Kong University of Science and Technology, Kowloon, Hong Kong China; 2 HKUST Jockey Club Institute of Advanced Study, The Hong Kong University of Science and Technology, Kowloon, Hong Kong China

**Keywords:** Computational science, Ultracold gases, Quantum simulation

## Abstract

The power of machine learning (ML) provides the possibility of analyzing experimental measurements with a high sensitivity. However, it still remains challenging to probe the subtle effects directly related to physical observables and to understand physics behind from ordinary experimental data using ML. Here, we introduce a heuristic machinery by using machine learning analysis. We use our machinery to guide the thermodynamic studies in the density profile of ultracold fermions interacting within SU(*N*) spin symmetry prepared in a quantum simulator. Although such spin symmetry should manifest itself in a many-body wavefunction, it is elusive how the momentum distribution of fermions, the most ordinary measurement, reveals the effect of spin symmetry. Using a fully trained convolutional neural network (NN) with a remarkably high accuracy of ~94% for detection of the spin multiplicity, we investigate how the accuracy depends on various less-pronounced effects with filtered experimental images. Guided by our machinery, we directly measure a thermodynamic compressibility from density fluctuations within the single image. Our machine learning framework shows a potential to validate theoretical descriptions of SU(*N*) Fermi liquids, and to identify less-pronounced effects even for highly complex quantum matter with minimal prior understanding.

## Abstract (web summary)

The detection of the effects of spin symmetry in momentum distribution of an SU(N)-symmetric Fermi gas has remained challenging. Here, the authors use supervised machine learning to connect the spin multiplicity to thermodynamic quantities associated with different parts of the momentum distribution.

## Introduction

Multi-component fermions with SU(*N*)-symmetric interactions hold a singular position as a prototype system for understanding quantum many-body phenomena in condensed matter physics, high-energy physics, and atomic physics ^1^. In condensed matter, e.g., interacting electrons usually possess SU(2) symmetry, whereas there are emergent higher spin symmetries for the low-energy property of systems as the SU(4) symmetry in graphene due to the combination of spin and valley degrees of freedom^2^. In quantum chromodynamics, nuclear interactions are represented by SU(3) symmetry^3,4^. In the past decades, developments in cooling and trapping of alkaline-earth-like fermions^5^ have opened possibilities to achieve even higher spin symmetries, owing to their distinctive inter-particle interactions, and thus provided ideal platforms to study various SU(*N*) fermionic systems^1,6,7^. Although the role of SU(*N*) symmetry has been probed in optical lattices^8–15^, the comprehensive characterization of interacting SU(*N*) fermions in bulk, wherein the SU(*N*) Fermi liquid description is valid, has still remained challenging^16–19^. One of the bottlenecks is that the interaction-induced effect enhanced by enlarged SU(*N*) symmetry is sufficiently pronounced by the tight confinement only in one-dimensional (1D)^16^ or two-dimensional (2D) cases^18^. It is only recently that thermodynamics and contact interactions are investigated in three-dimensional 3D SU(*N*) fermions^17,19^, but a comprehensive experimental study of SU(*N*) fermions still remains to be done. Developing experimental techniques or designing approaches to uncover the subtle connection of various spin multiplicity-dependent properties with the available experimental measurements in SU(*N*) interacting fermions is crucial to advance our understanding of SU(*N*) symmetry and the corresponding many-body phenomena.

Here we propose a framework to use machine learning (ML) as a guidance for the image analysis in quantum gas experiments and demonstrate the thermodynamic study of SU(*N*) fermions. The main idea of this heuristic approach can be summarized into a three-step process as follows: (1) manually control the amount of information within each of the images we feed to the neural networks (NNs) during the training or testing processes; (2) determine the relative importance of the given (or removed) information based on the changes in the accuracy of the training or testing processes; and (3) identify the connection between the information and specific physical observables, which we can further focus our analytical efforts on.

To demonstrate the proposed machinery concretely, we take a density profile of SU(*N*) Fermi gases as an example and show how it can guide the analytical studies. Besides the pronounced effects such as atom number and fugacity, we explore the connection between the spin multiplicity and less-pronounced features, such as compressibility and Tan’s contact in the density profile. Based on this machinery, we demonstrate that one can extract less-pronounced effects even in the most ordinary density profiles and we successfully reveal thermodynamic features, which depend on the spin multiplicity, from density fluctuations and high-momentum distributions. This allows one to detect the spin multiplicity with a high accuracy ~94% in a single snapshot classification of SU(*N*) density profiles. To further verify the validity of the connection between the less-pronounced effects and physical observable, we further measure the thermodynamic compressibility from density fluctuations within a single image benchmarking ML processes, which turns out to be in very good agreement with SU(*N*) Fermi liquid descriptions. Besides providing general-purpose methods to extract various less-pronounced effects and consolidate our understanding of SU(*N*) fermions, our approaches also complement recent ML studies of quantum many-body physics to explore the underlying physics^20–26^.

## Results

### Train NNs to classify SU(*N*) fermions

We begin by preparing the experimental measurements with appropriate labels. Here we choose one of the most ordinary experimental measurements for studying SU(*N*) Fermi gases, the density profile, and the spin multiplicity as the labels. In our experiment, a degenerate SU(*N*) Fermi gas with *N* = 1,2,5,6 is prepared in an optical trap and the density profile is recorded by taking spin-insensitive absorption images after time-of-flight expansion, yielding the momentum distribution. The spin multiplicity is confirmed by optical Stern Gerlach measurements (see “Methods”). In principle, the density profile contains the momentum-space information of SU(*N*)-interacting fermions, which reflects various thermodynamic observables, such as Tan’s contact or the compressibility, which is the underlying reason for the success of using ML techniques to detect the spin multiplicity. However, the effect of spin multiplicity on the momentum distribution is small compared to other features such as the fugacity and atom number because of small interaction strength. Therefore, the dataset should be prepared in such a way that images are indistinguishable based on the pronounced features (i.e., atom number or temperature), which forces the NN to seek for less-pronounced features. We post-select datasets and minimize possible correlations between spin multiplicity and atom number or temperature.

**Fig. 1:**
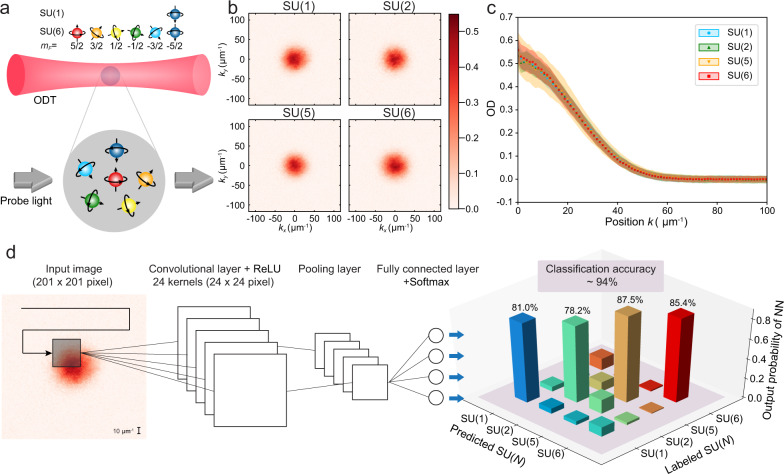
Distinguishing SU(*N*) fermions based on spin multiplicity by machine learning. **a** Schematic of preparing SU(*N*) gases in the optical dipole trap (ODT). The momentum distribution of the SU(*N*) Fermi liquid of ^173^Yb atoms with tunable spin configuration is recorded. The collected datasets are then fed into the NN as the input images for classification. **b** Examples of single experimental images of SU(*N*) gases. **c** Radially averaged optical density (OD) profiles in different SU(*N*) gases. The shaded region represents the fluctuation of the density profiles. **d** Experimental images of SU(*N*) gases are loaded into the neural network with one single convolutional layer. The black line and window represent how the kernel slides across the image. The output layer classifies the image into one of the class (i.e., SU(1), SU(2), SU(5), SU(6)) resulting in a classification accuracy around 94%. For each input image, NN outputs probabilities of different SU(*N*) with the highest value of the correct class. The output probabilities of NN are averaged over the test dataset.

In detail, we focus on the density profiles with the interaction parameters *k*_F_*a*_s_ ≃ 0.3 where *k*_F_ is the Fermi wave vector and *a*_s_ the scattering wavelength, and we only select the profiles based on similarities in widths of Gaussian fitting of the density profiles to result in indistinguishable momentum profiles as shown in Fig. 1b, c (see “Methods”). We collect 200 density profiles for each class of SU(1), SU(2), SU(5), and SU(6) (Fig. 1b). We randomly feed 150 of them to train the NNs by implementing the supervised ML techniques with spin multiplicity as labels and use the remaining 50 profiles to evaluate the classification accuracy that is defined as the ratio of number of samples with predictions matching true labels to the total sample number. To maximize the accuracy of NNs, we choose the architecture of convolutional NNs (CNNs) that is suited to explore the less-pronounced effects in an image (more details in “Methods”), as shown in Fig. 1d. By choosing the suitable structures and parameters in the CNNs, we can realize a very high accuracy ~94%, which is much better than the random guess (25%). We also test various unsupervised learning techniques such as the typical principal component analysis^27,28^ and only get a low classification accuracy of only ~43%. Moreover, it is worth to emphasize that the remarkably high accuracy ~94% of NNs is achieved by using only a single snapshot of the density profiles. All these results indicate that there are detectable, less-pronounced features in a single snapshot of density profile.

### Extract less-pronounced effects in low- and high-momentum parts

**Fig. 2:**
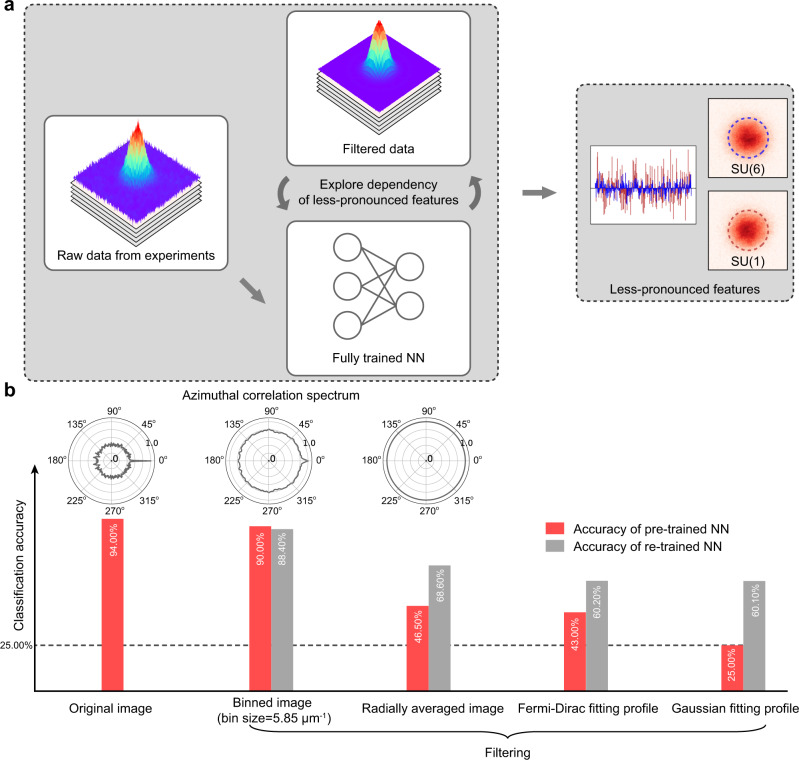
Exploring less-pronounced effects with NNs using deliberately filtered experimental images. **a** Our heuristic machinery to uncover less-pronounced effects. **b** Effect of image filtering on the classification accuracy by neural networks trained with original (red) and filtered (gray) experimental images. The retraining process follows the same procedure of original training with fixed neural network parameters and hyper-parameters (e.g., the number of epochs and learning rate). In the inset, the azimuthal correlation spectrums calculated at *k* = 58.5 μm^−1^ are shown.

We now analyze the attributes processed by the well-trained NNs and extract less-pronounced effects determined by the spin multiplicity step by step. Due to the limited interpretability of NNs, it is usually difficult to identify what kinds of features the NNs use for classification. In our proposed machinery, we examine which parts of the density profile are related to the spin multiplicity as described in Fig. 2a. Usually, it is more efficient to use some prior knowledge, which can be obtained in our limited understanding of the current system or the well-established understanding of the similar system. In our example of studying the interacting SU(*N*) fermions, we use the prior knowledge of non-interacting fermions and the associated energy (length) scale in choosing various filters in the momentum space. It is conceivable that our heuristic machinery can be applied to other systems.

To do this, we manually manipulate the experimental images and subsequently check the classification accuracy of the manipulated images. As different information is removed in different types of manipulated images, the classification accuracy will decrease with different amount, which will unfold what kind of information is more important for classification. As shown in Fig. 2b, we first replace the whole image with the Gaussian and Fermi–Dirac fitting profile to do the test based on the prior knowledge of non-interacting fermions. It turns out that the classification accuracy significantly decreases for both cases and the accuracy drop of the Gaussian fitting profile is even more, which implies the Fermi–Dirac type preserves the characteristics of the original profile better than the Gaussian fit. We further test the variations in accuracies by replacing profiles with radially averaged profiles, which results in test accuracies higher than the Fermi–Dirac fitting cases. However, the differences in accuracies between the radially averaged and Fermi–Dirac are much smaller compared to the differences between Fermi–Dirac and Gaussian, suggesting the SU(*N*)-dependent modifications of the Fermi–Dirac distribution to be small.

**Fig. 3:**
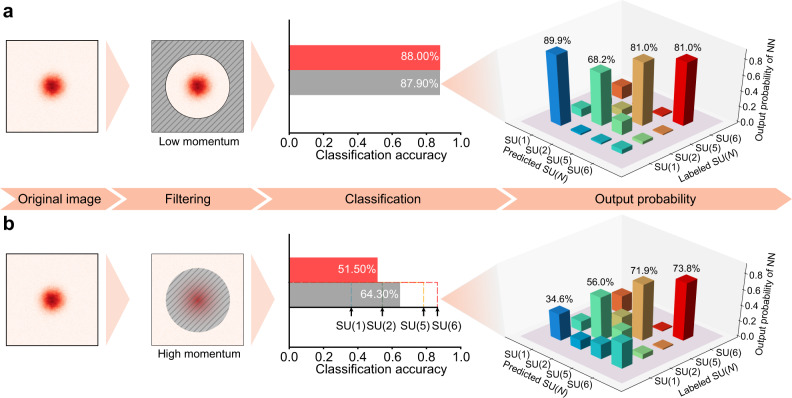
Influence of low- and high-momentum parts on classification. Classification accuracy with neural networks trained only with low **a** and high **b** momentum parts. Red and gray bars represent the accuracy of pre-trained and re-trained NNs, respectively. Retraining of the neural networks with only high- or low-momentum parts enhances the accuracy as shown in gray bars. For high-momentum parts, classification accuracy increases with *N* as shown in black ticks in **b**, where the average of the four investigated SU(*N*) cases is 64.30%. For the re-trained NN, the output probabilities of the NN are shown for input images.

Now we examine the contributions of low- and high-momentum parts by classifying the masked images with the well-trained NN as shown in Fig. 3a, b. This is motivated by the observation that the classification accuracy significantly decreases with filters, with various fitting functions that remove the SU(*N*)-dependent effect in the high-momentum tail. We choose two different types of masks for the region of the replacements, which will be referred as background and central masks, respectively. Background mask covers from the edge of the image to some atomic momentum *k*_c_, whereas the central mask covers from the center to *k*_c_. Then, we replace the masked region with a fake image generated by averaging the corresponding region of all the images in the dataset and re-evaluate the test accuracies of the pre-trained NN.

First, we set the cutoff momentum of *k*_c_ = 70 μm^−1^ such that >99% of atoms are contained within the low-momentum region (Fig. 3a), which still allows us to classify the spin multiplicity with the accuracy of 88%. Although this confirms that a NN perceives spin multiplicity-dependent information in low-momentum parts, questions remain on why the accuracies are not fully recovered beyond 94%. Such observation confirms the importance of the high-momentum information. In Fig. 3b, we prepare a dataset with a high-momentum part only (*k* > *k*_c_ = 70 μm^−1^), in which a low-momentum region is deliberately replaced by the same fake image. Surprisingly, the test classification accuracy is still >50% and the overall classification accuracy increases to 65% when a NN is re-trained. Such a high accuracy based on the few information of only <1% of atoms strongly implies that the high-momentum tail is crucial for determining the spin multiplicity. This SU(*N*)-dependent feature is not due to the finite resolution of the imaging system^29^, as the NNs can classify the binned image with high classification accuracy (see “Methods” for more information).

### High-momentum tails

In light of these results, we speculate that NNs utilize less-pronounced effects in the high-momentum part. To confirm this, we check the dependence of classification accuracy with the fully trained NNs on each SU(*N* = 1,2,5,6) class and we find the classification accuracy increases with *N* in Fig. 3b. In addition, the output probability of the correct spin multiplicity increases with *N* (see Fig. 3b). These results indicate that the less-pronounced feature being used in NNs becomes more prominent with increasing spin multiplicity *N*, which is consistent with the fact that atom–atom interactions are absent in the case of SU(1) due to the Pauli principle in the ultracold regime, while they are significantly enhanced in SU(6) fermions. Indeed, the amount of short-range interactions should be revealed in the high-momentum distribution in which the weight of such high-momentum tail is determined by the central quantity, so-called the contact^30–32^, in a dilute quantum gas. The contact governs many other physical observables^33,34^ and has been probed in strongly interacting gases^33–39^, and even in a weakly interacting gas with SU(*N*) symmetric interactions^17^. It is conceivable that the NNs detect the high-momentum distribution within a single image in contrast to the previous work where hundreds of momentum-space images are statistically averaged in a ^173^Yb Fermi gas^17^. To be noted, our observation is consistent with the direct measurement of the high-momentum tail in the region of *k*/*k*_F_ > 3^17^, which is corresponding to *k* > 100 μm^−1^ for an SU(1) gas in this work.

### Evaluating detection accuracy with tunable masks

**Fig. 4:**
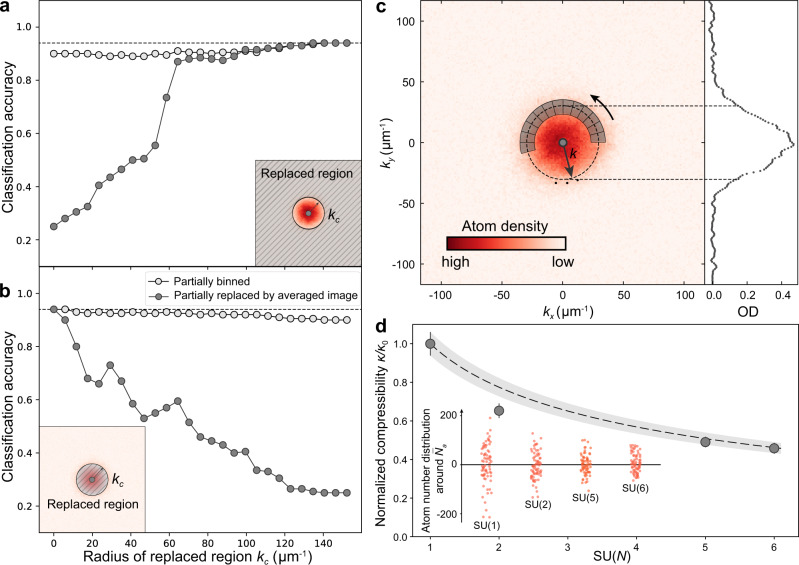
Verification of ML-aided detection : density fluctuations and thermodynamic compressibility. **a**, **b** Classification accuracy of the correct class as a function of the cutoff momentum *k*_c_ of the mask. The dotted line indicates the accuracy of 94%. **c** Measurement of density fluctuations with a snapshot. In a line-of-sight integrated density profile, a series of bins containing on average ${\overline{N}}_{a}$ atoms are chosen along the azimuthal direction. Each bin is about 10 (in azimuthal direction) by 17 (in radial direction) µm^−1^, which is much larger than the optical resolution of the imaging system. The density profile at *k*_x_ = 0 is shown. **d** The normalized compressibility of SU(*N*) fermions *κ*/*κ*_0_ is measured by relative density fluctuations as *κ*/*κ*_0_ = *ζ*_SU(*N*)_/*ζ*_SU(1)_. The error bar shows the SE. The dashed line indicates the theory curve $\kappa /{\kappa }_{0}={[1+\frac{2}{\pi }{k}_{\mathrm{{F}}}{a}_{\mathrm{s}}(N-1)(1+\epsilon {k}_{\mathrm{F}}{a}_{\mathrm{s}})]}^{-1}$ with the uncertainty represented by the shaded region considering the SE of *ζ*_SU(1)_. The inset shows the distribution of the atom number per bin from three images for each spin multiplicity. The distribution is plotted around the average normalized by the degenerate temperature, $(N-{\overline{N}}_{\mathrm{a}})/(T/{T}_{\mathrm{F}})$, where ${\overline{N}}_{\mathrm{a}}$ is the average atom number.

To examine the less-pronounced effects in both the low-momentum and high-momentum regions, and build up the concrete connections between these less-pronounced effects and the spin multiplicity, we now quantitatively analyze the changes inflicted on the test accuracies when the cutoff momentum *k*_c_ is tuned over. It is clearly shown in Fig. 4a that the test accuracy decreases to ~25% by complete replacements of the images by the same average image. This accuracy is gradually recovered up to almost 90% when *k*_c_ is increased to *k*_c_ ≃ 70 μm^−1^ being consistent with the result in Fig. 2, as less information from the low-momentum regime is removed. Based on replacement analysis, it is conceivable that SU(*N*)-dependent features must exist in the low-momentum part, as we will discuss the details below. In comparison, the classification accuracy of the binned images (bin size = 5.85 μm^−1^) does not decrease too much, which is only a partial removal of fluctuations. As a complementary study, we utilize the central mask and replace the low-momentum information up to a variable momenta of *k*_c_ in Fig. 4b. The classification accuracy gradually decreases with increasing *k*_c_ from 0 to ~50 μm^−1^, as the information within the density profile is increasingly removed. However, the classification accuracy stays over 50% around *k*_c_ = 50 ~ 70 μm^−1^, which strongly suggests that the high-momentum tails of the density distribution still contribute towards the classifications based on SU(*N*). Beyond *k*_c_ = 80 μm^−1^, where the atomic shot-noise becomes comparable to the background shot-noise level, the test accuracy rapidly drops for the images replaced by averaged images.

### Density fluctuations and compressibility

The question still remains as to what dominant feature classifies spin multiplicity in the low-momentum regime. Based on the significant decrease of the accuracy with profiles being radially averaged in Fig. 2b, we hypothesize that the NNs utilize the density fluctuation along the azimuthal direction for classification. The amount of azimuthal density fluctuations can be revealed in the correlation spectrum (Fig. 2b) showing a strong signal in the original and binned images, while flattened at all angles for the radially averaged images.

To understand how the density fluctuations reveal spin multiplicity, we consider the fluctuation–dissipation theorem by which the thermodynamic compressibility $\kappa =\frac{1}{{n}^{2}}\frac{\partial n}{\partial \mu }$ can be measured through density fluctuations (i.e., atom number fluctuations)^19,29,40,41^ where *n* is the local density and *μ* the local chemical potential. For repulsively interacting SU(*N*) fermions, it is known that the compressibility *κ* decreases with increasing spin multiplicity *N* as ${(\kappa /{\kappa }_{0})}^{-1}=1+\frac{2}{\pi }({k}_{\mathrm{F}}{a}_{\mathrm{s}})(N-1)(1+\epsilon {k}_{\mathrm{F}}{a}_{\mathrm{s}})$, where *κ*_0_ is the compressibility of an ideal Fermi gas and $\epsilon =\frac{2}{15\pi }(22-4\mathrm{ln}\,2)$^42^. Here, the atom number fluctuations are further suppressed by the Pauli blocking in the degenerate regime showing sub-Poissonian fluctuations as ${\sigma }_{{N}_{\mathrm{a}}}^{2}/\overline{{N}_{\mathrm{a}}}\propto {k}_{\mathrm{B}}T$ where *N*_a_ indicates the atom number measured in the small volume. Therefore, one finds the relative atom number fluctuation ${\sigma }_{{N}_{\mathrm{a}}}^{2}/\overline{{N}_{\mathrm{a}}}$ is given as

1 $${\sigma }_{{N}_{\mathrm{a}}}^{2}/\overline{{N}_{\mathrm{a}}} 	=n{k}_{\mathrm{B}}T\kappa \\ 	=\frac{3}{2}\frac{T/{T}_{\mathrm{F}}}{1+\frac{2}{\pi }{k}_{\mathrm{F}}{a}_{\mathrm{s}}(N-1)(1+\epsilon {k}_{\mathrm{F}}{a}_{\mathrm{s}})}$$

In our experiment, an atomic sample ballistically expands from the harmonic trap preserving occupation statistics of the phase space during the expansion^43,44^. Instead of repeatedly producing identical samples and monitoring a small region at the certain position^29^, the relative atom number fluctuations can be extracted along the azimuthal bins containing the same number of atoms on average (therefore, resulting in equivalent optical density) within a single image, even though a grouping of ideally equivalent bins is challenging and the fluctuation measurement is susceptible to the systematic variations. The successful classification of the spin multiplicity with NNs now guide us to subsequently investigate the atom number fluctuations with conventional analysis.

To verify this less-pronounced feature, we choose a series of bins containing ~450 atoms on average in a line-of-sight integrated density profile along the azimuthal direction (Fig. 4c) and analyze the statistics. To have a sufficiently large number of bins for statistical analysis, we perform the measurements at varying momenta on the ring with ${({k}_{\mathrm{x}}^{2}+{k}_{\mathrm{y}}^{2})}^{1/2}\simeq 28\;$ µm^−1^ around the center of the distribution. Both the temperature and spin multiplicity are known to affect the atomic-density fluctuations through the change in compressibility. Therefore, we normalize relative atom fluctuations by the temperature as ${\zeta }_{\text{SU}(N)}=\frac{{\sigma }_{{N}_{\mathrm{a}}}^{2}}{\overline{{N}_{\mathrm{a}}}}/\frac{T}{{T}_{\mathrm{F}}}$ to reveal SU(*N*) interaction effect from a single snapshot. In Fig. 4d, we then plot the statistical value of *ζ*_SU(*N*)_/*ζ*_SU(1)_. This measurement indeed reveals the normalized thermodynamic compressibility *κ*/*κ*_0_ = *ζ*_SU(*N*)_/*ζ*_SU(1)_ showing the enhanced interaction *k*_F_*a*_s_(*N* − 1). The error bar indicates the SE from 150 different density profiles. Whereas the scaling of the measured density fluctuation with *N* is in good agreement with theoretical prediction, experimental results for SU(*N* > 1) lie systematically below theoretical ones. The discrepancy may be due to interactions that remain finite during the expansion, which could slightly perturb the occupation statistics of the phase space. Considering the fact that the change of the compressibility is not significant for *N* = 5 and 6 in Fig. 4d, the high classification accuracy of NNs using the low-momentum part highlights the superior capabilities of a single snapshot approach using ML. In contrast to the conventional analysis that focuses on a single observable, NNs take a holistic approach in utilizing multiple features simultaneously. Our measurement is consistent with recent experiments in which thermodynamics is studied by monitoring the density fluctuations and expansion dynamics in degenerate ^87^Sr atoms^19^.

## Discussion

To scrutinize the effects of SU(*N*) symmetric interactions, we have provided the NN with altered images and probed specific attributes of the profiles independently. We found that the high-momentum tail and density fluctuation information significantly contribute to the SU(*N*) classification process. First of all, the high-momentum tails of atomic-density distributions are expected to exhibit Tan’s contact, which encapsulates the many-body interactions through the set of universal relations. Although the previous work required averaging of hundreds of images for the detection of the SU(*N*)-dependent contact^17^, the NN’s ability makes it possible to obtain the single-image distinguishability of the SU(*N*) class after training. However, the exact mechanism behind how the trained network collects the required information for extraction of the contact, whether it is through superior noise suppression or signal enhancement, is not known and is left for future work. Furthermore, it is conceivable that the regression algorithms can be used to extract the change of contact for different spin multiplicity in future works.

The second dominant feature for the SU(*N*) classification is the density fluctuation within the profile. Both the temperature and spin multiplicity are known to affect the atomic-density fluctuations through the change in compressibility. Sub-Poissonian density distributions have been observed in degenerate Fermi gases of atoms^29,40^ and molecules^41^, where multiple images were used to obtain the statistics. The suppression of the density fluctuation was also observed in SU(*N*) fermions allowing for the thermodynamic study^19^. For a single image, there exists multiple sets of density fluctuation measurements at varying momentum, where each measurements form a ring around the center of the distribution. Considering the decreased SU(*N*) classification accuracy from the radially averaged datasets, the fluctuation information might have been utilized in addition to the contact, to reflect the effects of compressibility. Lastly, we found that the low-energy part of the density profile does not exhibit a signature as strong as the previous two features. Although there has been a report of SU(*N*)-dependent modifications to the density distribution limited to the 1D case^16^, the corresponding beyond mean-field effects in 3D remains challenging to be measured experimentally.

In conclusion, we have demonstrated the capabilities of the proposed machinery by classifying SU(*N*) Fermi gases with their time-of-flight density distributions. The NN provides classifications with an accuracy well beyond the conventional methods such as principal components analysis. By applying different types of manipulations, we also find that the NNs combine the features from a high-momentum signal and density fluctuations together, to distinguish SU(*N*). Future directions include predictions of *T*/*T*_F_ of SU(*N*) Fermi gases based on regression algorithms and explorations of human feedbacks to the ML process for feature extractions. Feature extraction through ML may guide us to investigate the right information and facilitate research in many-body quantum systems.

## Methods

### Sample preparation

We prepare a balanced ultracold Fermi gas of ^173^Yb atoms with SU(*N*) symmetric interactions as large as *N* = 6. (shown in Fig. 1a). We begin by loading a laser-cooled, six-component Fermi gas, where the nuclear spin states are equally populated, into a three-dimensional optical dipole trap (ODT). The atoms are further evaporatively cooled in the ODT to a temperature range of 0.2–1.0 *T*/*T*_F_, where *T*_F_ is the Fermi temperature. During the evaporation, different spin configurations are prepared via an optical pumping process using a narrow line-width transition of ^1^*S*_0_(*F* = 5/2) → ${\,}^{3}{P}_{1}(F^{\prime} =7/2)$ at a wavelength of *λ* = 556 nm. The *σ*^±^-polarized pumping light removes unwanted *m*_F_ states of the ground manifold of ^1^*S*_0_^45^ and produces a Fermi gas with tunable SU(*N*) interactions, as the nuclear spin relaxation rates are negligible in our experiment. After the evaporative cooling, the ODT is further ramped up in 60 ms, to obtain large-enough trap frequencies (*ω*_x_, *ω*_y_, *ω*_z_) = 2*π* × (1400, 750, 250) Hz before 4 ms of time-of-flight expansion. We measure the density distributions by taking absorption images using a spin-insensitive ^1^*S*_0_(*F* = 5/2) → ${\,}^{1}{P}_{1}(F^{\prime} =7/2)$ transition at 399 nm. The images are taken in random order with respect to their spin configurations, to avoid the possibility of a classification based on fluctuations in the background. The spin configuration of the sample can be monitored by the optical Stern Gerlach measurement. In general, the atom number of different spin states has a fluctuation of ±2% of the total atom number.

### Data preparation

All snapshots are first preprocessed by the fringe removal algorithm reported in ref. ^46^. Then, cropped images are loaded into the NN for further classification. For SU(*N*) data, it is natural to prepare the same number of atoms per spin at constant *T*/*T*_F_, in which the normalized density profile is the same for different SU(*N*) cases. In this case, however, we find that the diffraction of the imaging light induces fringe patterns that depend on the total atom number in the experiment. One can normalize the image by the total atom number, but we inevitably change the level of background noise. Therefore, we keep the total atom number unchanged, otherwise the NN uses the background fringe patterns or noises to classify the SU(*N*) data. In our experiment, we post-select 200 images per each SU(*N*) class by using a Gaussian fitting, which allows us to obtain samples with similar profiles but different *T*/*T*_F_. If we have kept different SU(*N*) gases at constant *T*/*T*_F_, the profiles are identical in the unit of *k*_F_, instead of pixel. Subsequently, 75% of the data is used for training NNs and the remaining is for test.

### Machine learning

ML, a sub-field of artificial intelligence, allows us to understand the structure of data and deduce models that explain the data. Traditionally, ML can be classified into two main categories, supervised and unsupervised learning, based on whether there are labels or not for training. Supervised learning usually trains a model from a known dataset of input data {*x*_*i*_} and output labels {*y*_*i*_} so that the model can find a correspondence rule *x*_*i*_ ↦ *y*_*i*_, which allows us to predict the labels of data beyond the training dataset. In contrast, unsupervised learning is used to classify the data into several different clusters based on the potential patterns or intrinsic structures in the dataset without any prior knowledge of the system or data properties.

### Convolutional neural network

ML techniques used in this work are based on CNNs, which takes a supervised learning approach for classification task. NNs, inspired by the biological NNs that constitute animal brains, are composed of a series of artificial neurons, among which the connection is a real-valued function $f:{{\mathbb{R}}}^{k}\to {\mathbb{R}}$, parameterized by a vector of weights $({w}_{1},{w}_{2},...,{w}_{i},...)={\bf{w}}\in {{\mathbb{R}}}^{k}$ and the activation function $\phi :{\mathbb{R}}\to {\mathbb{R}}$, given by

2 $$f(x)=\phi ({\bf{w}}\cdot {\bf{x}})\quad {\rm{with}}\quad {\bf{x}}=({x}_{1},...,{x}_{i},...)\in {{\mathbb{R}}}^{k}.$$

By combining the artificial neurons in a network or in a layer of network, we obtain NNs. In recent years, CNNs have shown stronger validity and better performance than regular NN in image recognition. Similar to the regular NNs, CNNs also consist of a sequence of layers and each layer receives some inputs, performs a dot product, and optionally follows it with a nonlinear activation function. However, unlike a regular NN, a CNN usually has several convolutional layers where neurons are arranged in two dimensions, providing an efficient way of detecting the spatial structure. The convolutional layer first accepts an input 2D image from the previous layer or the whole NN. Then, the kernel of the convolutional layer slides (i.e., convolve) across the width and height of the input image, with dot products between the kernel and the input being computed. Consequently, we obtain a 2D feature map in which each pixel is the response at the corresponding position. If the convolutional layer has *N* different kernels, the same procedure will be repeated for each kernel and finally *N* 2D feature maps will be produced. These 2D feature maps will then be loaded into the next layer as input.

### Training and evaluating the NN

The CNNs used in this study are realized by using the Tensorflow in Python^47^. We have attempted different architectures and found that the result is not sensitive to the choice of architecture such as the number of layers or the kernel size. Therefore, we remove superfluous layers to simplify our model. The concrete parameters taken in this work are listed in Table 1.

**Table 1:** Network architecture and parameters used in this work.

Layer	Layer name	Function	Description
1	Input	Image input	201 × 201 Images
2	Conv. layer	Convolution	24 24 × 24 Convolutions with stride (1,1)
3	ReLU	Activation function	ReLU function
4	Pool layer	Average pooling	2 × 2 With stride (1,1)
5	Dropout	Dropout	50% Dropout
6	Fully conn. layer	Fully connected	Fully connected layer with 4 neurons
7	Softmax	Activation function	Softmax function
8	Output	Classification output	Probability with classes *N*=1,2,5,6

To train the network on the data with different spin configurations, the model is compiled with a cross-entropy loss function. During the training process, the weights of model are updated based on Adam algorithm^48^, to minimize the loss function with a learning rate of 1 × 10^−4^, which is a hyper-parameter that controls how much the network changes the model each time. The maximum training epochs are limited to 1000, and the accuracy and loss are monitored during the training process for selecting the model with best performance. After full training, we evaluate the trained model on the test dataset.

We characterize the performance of trained NNs by obtaining the overall classification accuracy, which is defined as the ratio of number of samples with predictions matching true labels to the total sample number. For one single image loaded into the NNs, e.g., the softmax activation function normalizes the output values {*σ*_c_} by $P(c)={e}^{{\sigma }_{c}}/\mathop{\sum }\nolimits_{c = 1}^{4}{e}^{{\sigma }_{c}}$, which allows a probabilistic interpretation for the different classes denoted by the subscript *c*. When calculating the classification accuracy, the class with highest probability *P*(*c*) is selected as the prediction from NNs. As a complementary analysis of NNs, we also evaluate an output probability matrix that hints how the NNs perform among different classes, such as that shown in Figs. 1d and 3. In the probability matrix, every element *A*_*i*,*j*_ represents the probability *P*(*c*) averaged over the results for all images with the true label *j* and prediction *i*.

### Manipulation of SU(*N*) data

**Fig. 5:**
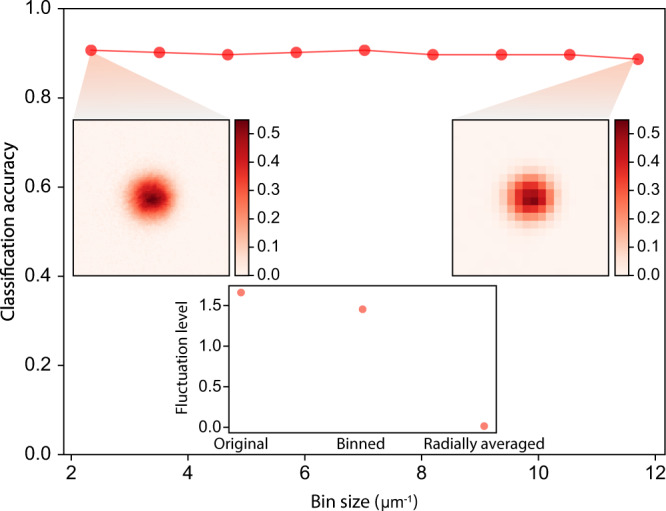
Classification accuracy with different bin sizes. The classification accuracy is quite robust when bin size *n* is changed from 2.3 to 11.70 μm^−1^. The inset shows the comparison of fluctuation level among the original image, binned image, and radially averaged image.

In this work, we manipulate the experimental images to remove different types of information. In Fig. 2, we examine the binned image, radially averaged image, Fermi–Dirac fitting profile, and Gaussian fitting profile. The blurring of adjacent pixels effectively changes subtle features in SU(*N*) gases due to finite optical resolution, e.g., it will decrease the measured atom number variance^29^. We minimize this effect by binning the data using a sufficiently large bin size^29^. The whole image is partitioned into bins with the area of *n* μm^−1^ × *n* μm^−1^ without overlapping. In each bin, the averaged optical density with the bin is calculated and the value is subsequently used to fill all the pixels of the bin to maintain the original size of image. We attempted several different bin size *n* from 2.34 to 11.70 μm^−1^ and the result is robust against the bin size, as shown in Fig. 5.

For the radially averaged images, we first divided all the pixels into several bins based on the distance from the center of the atom cloud, then averaged the pixels in the same bin. The degenerate Fermi–Dirac and Gaussian profiles are fitted by the 2D Thomas–Fermi and Gaussian distribution, respectively. It is worth noting that both density fluctuations and high-momentum information are effectively removed from both fitting cases. Therefore, the comparison between Fermi–Dirac and Gaussian profiles may allow one to investigate possible next-order effects by which NNs detect the changes in *T*/*T*_F_.

In Fig. 3, we first divide the whole image into two parts, low-momentum and high-momentum regions, based on whether the distance from the center of the atom cloud is larger than 70 μm^−1^ or not. Next, we replace one of the two parts with a fake image, which is generated by averaging the corresponding region of all the images (*N* = 1,2,5,6) in the dataset. As all the test images are same in the replaced region, the information in that region can be considered as removed. In Fig. 4a, b, the procedure is the same as in Fig. 3 with variable cutoff momenta. For the partially binned images, the corresponding region is replaced by a binned image.

### Data analysis

In Fig. 2a, we calculate the azimuthal correlation spectrum at *k* = 58.5 μm^−1^, which is defined as ${C}_{k}({\theta }_{j})=\frac{{\sum }_{i}[{P}_{k}({\theta }_{i}){P}_{k}({\theta }_{i+j})]}{{\sum }_{i}{P}_{k}^{2}({\theta }_{i})}$, where *P*_*k*_(*θ*_*i*_) represents the optical density for a specific pixel at *k* ~ 58.5 μm^−1^ and angle ~ *θ*_*i*_. The formula can be further derived from the Fourier transform ${\mathcal{F}}$ as $G=\frac{{{\mathcal{F}}}^{-1}(| {\mathcal{F}}({P}_{k}){| }^{2})}{| {P}_{k}{| }^{2}}$. The azimuthal correlation spectrum shows how the image looks like its own copy after rotating a specific angle. For a radially averaged image, the correlation becomes 1 at any angle, indicating no density fluctuations. When the image is binned, densities are only locally averaged resulting azimuthal correlation at nonzero angle < 1.

Figure 4d shows density fluctuations measured along azimuthal bins containing the same number of atoms within a single image. Total 24 bins are chosen at the distance of 22 ~ 34 μm^−1^ from the center of the cloud. Redundant pixels are removed at the border to keep all bins having the same number of pixels. The size of each bin is much larger than the optical resolution of the imaging system.

## Supplementary information

Supplementary Information

## Data Availability

The data that support the finding of this work are available from the corresponding authors upon request.

## Code availability

The code developed during the current study is available from the corresponding authors upon request.
