# Supplementary material for: Heuristic machinery for thermodynamic studies of SU(N) fermions with neural networks
Source: Nat Commun. 2021 Mar 31;12:2011. doi: 10.1038/s41467-021-22270-5 (PMC8012572; doi:10.1038/s41467-021-22270-5)
Supplement: Supplementary file 1 — Supplementary Information [file 41467_2021_22270_MOESM1_ESM.pdf]

SUPPLEMENTARY INFORMATION
for
"Heuristic machinery for thermodynamic studies of SU(N) fermions with neural networks"

Entong Zhao,[1] Jeongwon Lee,[2] Chengdong He,[1] Zejian Ren,[1] Elnur Hajiyev,[1] Junwei Liu,[1] and Gyu-Boong Jo[1]

[1]*Department of Physics, The Hong Kong University of Science and Technology, Clear Water Bay, Kowloon, Hong Kong, China*
[2]*HKUST Jockey Club Institute of Advanced Study, The Hong Kong University of Science and Technology, Clear Water Bay, Kowloon, Hong Kong, China*

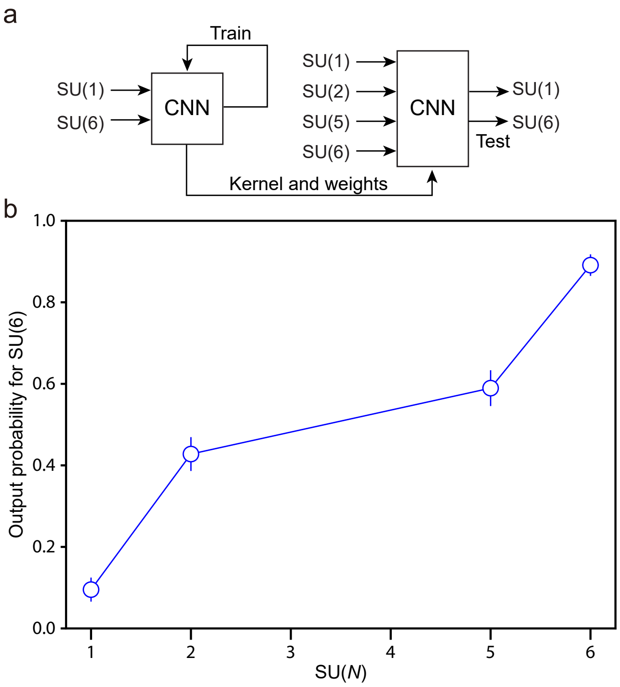

Supplementary Figure 1. **Continuity of classification variables. a** The neural network is trained to distinguish SU(1) and SU(6) fermions only. After the training process, all of the four classes are shown to the neural network for prediction. **b** For each SU(N) class, the CNN computes the probability that the test image can be classified into SU(6). The error bars represent the standard error of the 50 test images.

Supplementary Note 1: Continuity of classification variables

In contrast to the discrete classification variables used in the main text, we examine the training of the neural network with only SU(1) and SU(6) data, and use the trained network to evaluate a classification probability of a given test image from a larger pool of classes with N={1,2,5,6} (shown in Supplementary Fig.1a). We first train the neural network with 150 images per class for SU(1) and SU(6) resulting the validation accuracy over 95%. Then we use the trained neural network to evaluate all classes N={1,2,5,6}. The result in Supplementary Fig.1b shows that the NN tends to compute the SU(6) classification probabilities of SU(5) test images higher than SU(2). We also repeat this procedure for 20 times with the same network parameters in order to avoid an accidental effect, and the results from each independent process show similar tendency. This observation may imply that there exists a continuously varying observable for the neural network to determine the spin configuration, rather than a discrete mapping between the features and individual classes. However, it remains unclear to what extent one can interpret the physical meaning of the result. One possible model may be a regression algorithm which can predict a continuous value from input

features and may allow us to grasp the working principle based on a variable evenly distributed in the control variable space.

Supplementary Note 2: Unsupervised learning

KMeans cluster algorithm [1] is a common technique used in unsupervised classification tasks. By continuous iteration, KMeans method finds the center of different clusters and classifies the data according to the distance from the center. However, we found that the KMeans algorithm is not promising to classify the SU(N) data since the algorithm relies on a measure of similarity between the data points, which is ambiguous in our SU(N) data [2]. Therefore, we use the Principle component analysis (PCA), which is widely used for extracting features of the data, to find a low-dimensional representation of the data set before applying the KMeans algorithm. The PCA method converts a set of possibly correlated variables into a set of values of linearly uncorrelated variables, so-called principal components, through a linear transformation of the original coordinates, Usually the first few principle components contain most of the information, which makes PCA an efficient tool for dimensional reduction.

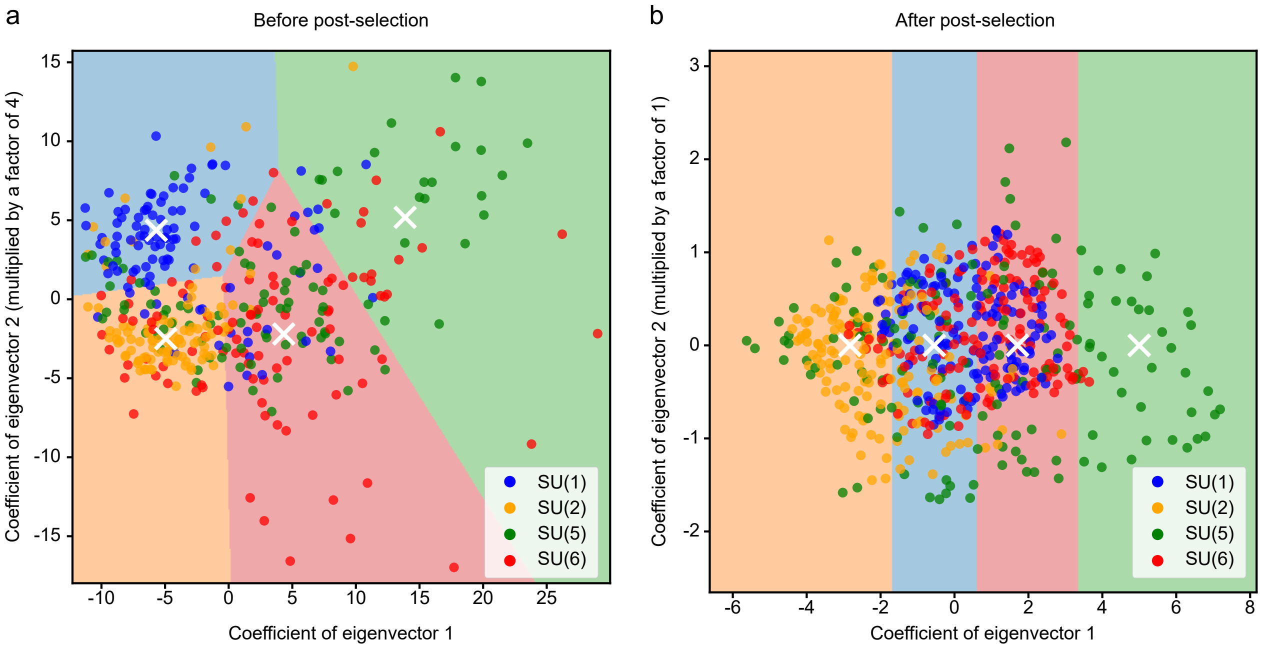

Supplementary Figure 2. **Comparison between results of unsupervised learning before and after post-selection.** **a** Results of unsupervised learning before post-selection. The whole space spanned by the first two eigenvectors is separated into four different areas filled with different colors based on the distribution of the samples. Each area represents the predicted region of one type of SU(N) gases. The second eigenvector is multiplied by a factor of 4 to realize the best performance and the classification accuracy is around 55.8%. The white crosses are the centers of the clusters. **b** Results of unsupervised learning after post-selection. The whole space spanned by the first two eigenvectors is separated into four different areas based on the distribution of the samples. Each area represents the predicted region of one type of SU(N) gases. The second eigenvector is multiplied by a factor of 1 to realize the best performance and the classification accuracy is around 43.5%. The white crosses are the centers of the clusters.

From the result of the KMeans cluster after PCA, overall classification accuracy is around 43.5 %. Lower accuracy of the PCA compared to the NN case implies that the lower dimensional information (likely to be more pronounced parameters such as the atom number and size of the cloud) of the images are insufficient for the classification process. On the other hand, we examine how the post-selection process suppresses correlations between pronounced effects and spin multiplicity by comparing the classification accuracy before (55%) and after post-selection (43%) in Supplementary Fig. 2.

Supplementary Note 3: Model visualization

To understand how the NN can make the classification, we visualize the weights of convolution kernels, activations of convolution kernels, weights of fully connected layers and overall activations of our trained NN in Supplementary

Fig.3. Unlike the case of analyzing visual imagery, it is difficult to see obvious patterns (e.g., lines, edges or more complicated patterns) in neither kernel weights nor activations. It could be attributed to that our images are similar in contrast to the common visual imagery, which suggests the overall activation region should be similar for different SU(N) fermions. Besides, our data is also quite smooth compared with the common visual imagery, and therefore it is not expected to have clear patterns like lines or edges.

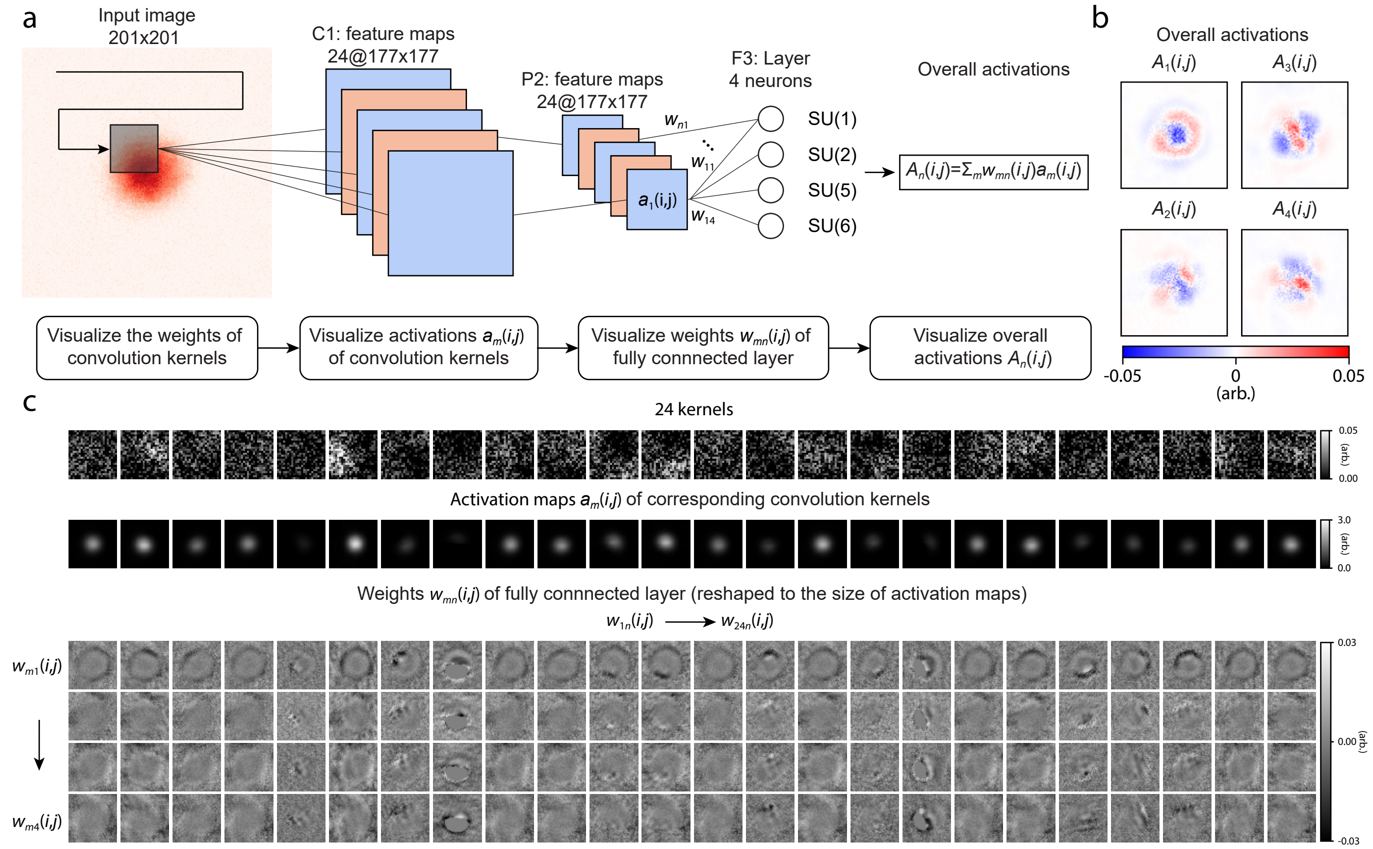

Supplementary Figure 3. **Visualization of the NN.** **a** We visualize the weights of convolution kernels, activations $a_m(i,j)$ of convolution kernels, weights $w_{mn}(i,j)$ of fully connected layers and overall activations $A_n(i,j)$, where overall activations for specific class n is defined as the dot product between the activations $a_m(i,j)$ and corresponding weights $w_{mn}(i,j)$ of fully connected layer at each pixel. **b** The overall activations $A_n(i,j)$ with $n=1,2,3,4$ representing the class SU(1), SU(2), SU(5), SU(6), respectively. **c** Visualization of the 24 kernels (first row), corresponding activation maps (second row) in the convolutional layer and weights of fully connected layer. The weights of fully connected layers are reshaped to the size of activation maps. The column represents the index of connected activation maps and the row represents the connected output class.

Supplementary References

[1] F. Pedregosa et al. Scikit-learn: Machine Learning in Python. *Journal of Machine Learning Research* **12**, 2825-2830 (2011).

[2] L. Wang. Discovering phase transitions with unsupervised learning. *Phys. Rev. B* **94**, 195105 (2016).
